# Analysis of Association between Dietary Intake and Red Blood Cell Count Results in Remission Ulcerative Colitis Individuals

**DOI:** 10.3390/medicina55040096

**Published:** 2019-04-08

**Authors:** Dominika Głąbska, Dominika Guzek, Barbara Kanarek, Gustaw Lech

**Affiliations:** 1 Department of Dietetics, Faculty of Human Nutrition and Consumer Sciences, Warsaw University of Life Sciences (SGGW-WULS), 02-776 Warsaw, Poland; barbara_kanarek@sggw.pl; 2 Department of Organization and Consumption Economics, Faculty of Human Nutrition and Consumer Sciences, Warsaw University of Life Sciences (SGGW-WULS), 02-776 Warsaw, Poland; dominika_guzek@sggw.pl; 3 Department of General, Gastroenterological and Oncological Surgery, Medical University of Warsaw, 02-097 Warsaw, Poland; gustaw.lech@wum.edu.pl

**Keywords:** ulcerative colitis, anemia, iron, energy, meat, case-control study

## Abstract

*Background and objectives:* The anemia is the most common extra-intestinal manifestation of the ulcerative colitis. Taking into account, that meat products are perceived as factor, that may promote relapses, the crucial is to indicate the dietary recommendations to prevent anemia development but without high animal products intake. Aim of the study was to analyze the influence of animal products intake on the red blood cell count results in remission ulcerative colitis individuals and pair-matched control group, during 6 weeks of observation. *Materials and Methods:* The intake of nutrients associated with anemia development (iron, vitamin B_12_, protein, animal protein, calcium) and the products being their sources (meat, meat products, dairy beverages, cottage cheese, rennet cheese) were analyzed. *Results:* In spite of the higher meat products intake in the group of ulcerative colitis individuals, the iron intake did not differ between groups. The positive correlations between intakes of meat, meat products, total protein, animal protein, iron, vitamin B_12_ and red blood cell count results were stated for ulcerative colitis individuals, while in control group were not observed, that may have resulted from higher susceptibility for the diet-influenced changes. *Conclusions:* The positive correlation between red blood cell count results and energy value of diet, and daily iron intake observed in ulcerative colitis individuals, accompanied by negative correlation for iron intake per 1000 kcal of diet, may indicate, that higher iron intake may be beneficial, but only while accompanied by high energy value of diet.

## 1. Introduction

The anemia is the most common extra-intestinal manifestation of the inflammatory bowel diseases-both ulcerative colitis and Crohn disease [1]. In the systematic review and individual patient data meta-analysis of Filmann et al. [2] it was indicated, that in the European countries, the overall prevalence of anemia in Crohn disease individuals is 27% and in ulcerative colitis individuals-21%. In spite of the fact, that a number of factors may contribute to high anemia frequency, including iron, vitamin B12 and folic acid deficiencies, iron deficiency is indicated as the most common reason in mentioned group [3]. Moreover, in spite of the fact, that the general prevalence of anemia in the inflammatory bowel diseases is not higher than 30%, the prevalence of iron deficiency is much higher, as it is observed in 60–80% of individuals [4].

The deficits of iron, vitamin B_12_ and folic acid observed in ulcerative colitis individuals are associated with a number of factors-insufficient intake, increased needs and excessive loss, that may result not only from the disease and its symptoms, but also from the applied treatment [5]. As a result, not only supplementation is recommended for individuals with deficits [6], but also a number of authors indicate the need for increasing intake of specific nutrients in comparison with recommended for the general population [7,8,9,10,11,12,13,14].

Taking into account, the fact, that both vitamin B_12_ and heme iron, that is characterized by higher absorption than non-heme iron [15], are derived from animal products [16], the intake of animal products may be crucial in anemia prevention in ulcerative colitis. However, in a number of studies, animal products were indicated as having both the negative impact in etiology and negative impact on the course of the inflammatory bowel diseases. It was stated, that increased animal protein intake is the strongest independent factor contributing to the Crohn disease development [17]. Simultaneously, the intake of meat, especially of red and processed meat, as well as intake of protein were indicated as associated with an increased risk of relapses in ulcerative colitis patients [18], as it promotes inflammation [19]. As a result, it may be stated, that in ulcerative colitis individuals, animal products intake should be as high as needed to provide the lowest anemia risk, but at the same time, should be as low as possible to reduce the risk of relapses.

Taking into account the fact, that a number of authors indicate, that anemia in inflammatory bowel disease individuals is a serious problem, that should be no longer neglected [3,20], the crucial is to indicate the most important dietary recommendations to maintain proper level of blood count results and prevent anemia development for ulcerative colitis patients but without too high animal protein intake.

The aim of the study was to analyze the influence of animal products intake on the red blood cell count results in remission ulcerative colitis individuals and pair-matched control group, during 6 weeks of observation.

## 2. Experimental Section

### 2.1. Study Design

The study was conducted at the Dietetic Outpatient Clinic of the Department of Dietetics, Warsaw University of Life Sciences (WULS-SGGW). During 6 weeks of observation, the 3-day dietary records and the red blood cell count results were simultaneously analyzed in remission ulcerative colitis individuals and pair-matched control group. The study was conducted according to the guidelines laid down in the Declaration of Helsinki and all procedures involving human subjects were approved by the Bioethical Commission of the National Food and Nutrition Institute in Warsaw (No. 0701/2015). Written informed consent was provided by all participants.

During the 6 weeks of observation, participants were assessed 4 times, while the interval of 2 weeks between the assessments was applied, due to the fact, that the red blood cell count results are in the constant flux, but the flux is limited by a lifespan of cells [21]. Before the study and during the study, participants did not receive any dietary counseling in order to avoid any additional external influence exerted on the diet.

**Figure 1:**
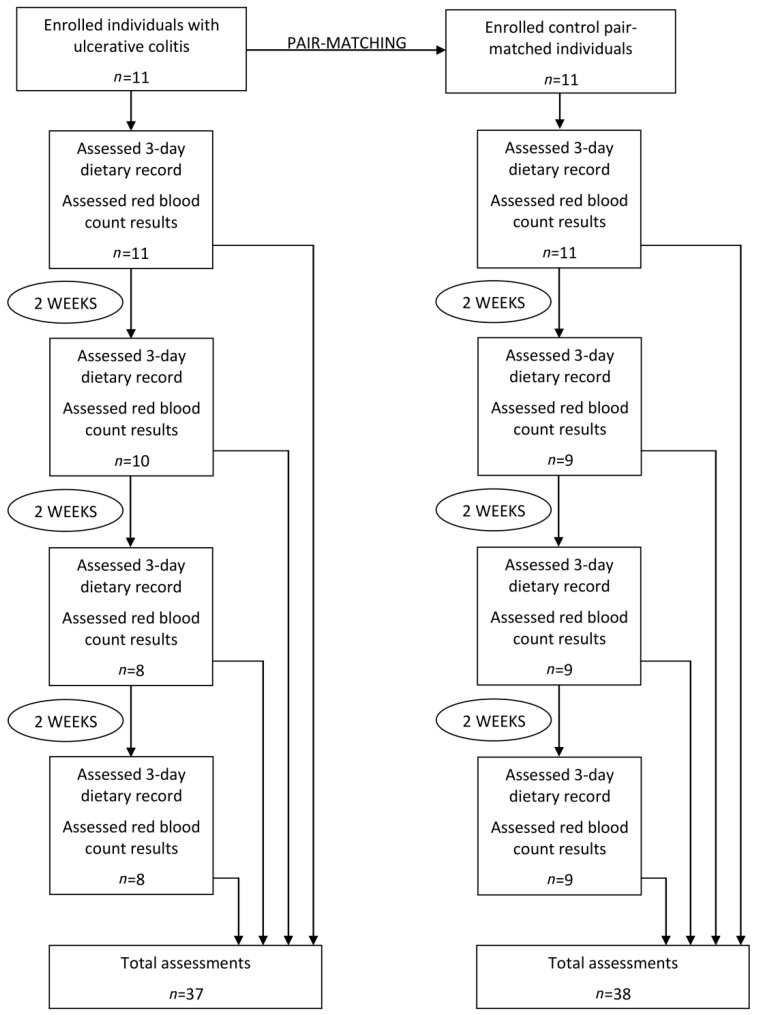
The study design and number of participants.

While during the study participant refused further participation in the study, he was excluded from the further assessments, but his results from previous assessments were included into analysis, as well as the pair-matched individual was not excluded. As a result, the number of assessments in ulcerative colitis group and in pair-matched control group are not equal, as in ulcerative colitis group, the total number of 37 assessments was conducted, while in control group-a number of 38 assessments. The study design and number of participants are presented in Figure 1.

### 2.2. Study Participants

The study was conducted among individuals with ulcerative colitis in remission, both males and females who were recruited and monitored at the following Warsaw Gastroenterology Outpatient Clinics: Gastroenterology Outpatient Clinic at Maria Skłodowska-Curie Memorial Cancer Centre, Institute of Oncology; Gastroenterology Outpatient Clinic at the Central Clinical Hospital of the Ministry of Interior in Warsaw and Gastroenterology Outpatient Clinic at the Public Central Teaching Hospital in Warsaw. The control group was pair-matched, taking into account the gender, age and the chronic diseases other than ulcerative colitis, as in the previously conducted study [22].

A total number of 11 individuals with ulcerative colitis in remission and a total number of 11 pair-matched control individuals were recruited for the study. Inclusion criteria for individuals with ulcerative colitis were the same as in the previously published studies [23,24,25]: free-living patients with endoscopically diagnosed ulcerative colitis and confirmed clinical remission (assessed on the basis of the Mayo Scoring System and the Rachmilewitz index for assessment of ulcerative colitis activity), accompanied by confirmed endoscopic remission (image with no changes or disappearance of the vascular network, erythema, inflammatory polyps allowed) if routine endoscopy was conducted during last 6 weeks, age 18–80 years, individuals characterized by clinical remission lasting at least 6 weeks, and a constant dose of drugs for at least 6 weeks. For the Mayo Scoring System, the cut-off of two points in a 12-points scale and for the Rachmilewitz index the cut-off of four points in a 31-points scale were chosen to recruit the remission individuals, as it is commonly indicated [26]. The inclusion criteria for the control group were: lack of inflammatory bowel diseases and pair-matching with individuals with ulcerative colitis (factors taken into account during matching were: gender, age ±2 years and the chronic diseases other than ulcerative colitis). The exclusion criteria for both groups were: previous extensive gastrointestinal resections (as a factor that may cause impaired digestion or absorption), cancer during previous 5 years (as a factor that may cause lower body nutrients reserves), pregnancy or breastfeeding (as a factor that may cause increased needs) and nutrients supplementation. All of the participants provided written consent to participate in the study.

### 2.3. Analysis of Diet

The assessments of diet were based on self-reported data from the patients’ dietary records conducted over a period of three typical, random days (2 weekdays and 1 day of the weekend). The dietary record was conducted on the basis of widely accepted and applied rules-using a structured format, with additional questions about name of the meal, time and location of consumption, meal ingredients and weight of serving (while weighted using kitchen scale) or size of serving (while estimated using standard household measures) [27]. To provide reliable estimates of food intake, participants were instructed on the principles of making the dietary record as well as on the necessity of accurate and scrupulous recording of all food products consumed and beverages drunk, while the serving sizes were verified afterwards by a dietitian using the Polish food model booklet [28].

The energy values of the diets were analyzed using Dieta Polish dietician software version 5.0 (National Food and Nutrition Institute, Warsaw, Poland) and the Polish base of the nutritional values of products [29]. The nutrients associated with anemia development and derived from animal products were included into analysis. Except from the iron and vitamin B12, directly associated with anemia development [3], the protein and animal protein, enhancing iron absorption [30], as well as calcium, inhibiting iron absorption [31], were analyzed. The nutrients derived from plant products were excluded from the analysis. The nutrients intake levels were presented as a daily intake and were recalculated per 1000 kcal of diet in order to analyze also the nutrient density of the diet.

The additional assessment of the sodium intake was conducted, and the additional analysis of the association was for sodium conducted, as it is indicated a dietary factor promoting colitis [19].

The consumed dishes declared in the 3-day dietary record were recalculated per food products, using the standard recipes of dishes [32], modified on the basis of information obtained from participants, by dietitian, during record verification. On the basis of obtained data, the intake of animal products being sources of nutrients associated with anemia development (meat, meat products, dairy beverages, cottage cheese, rennet cheese) were calculated and included into analysis. The products intake levels were presented as a daily intake and were recalculated per 1000 kcal of diet in order to analyze also the products density of the diet.

### 2.4. Measurement of the Red Blood Cell Count Results

The measurements of the red blood cell count results were conducted in a fasting state. During each measurement, the following parameters were assessed: red blood cells (RBC), haemoglobin (HGB), hematocrit (HCT), mean corpuscular volume (MCV), mean corpuscular hemoglobin (MCH), mean corpuscular hemoglobin concentration (MCHC), red blood cell distribution width (RDW).

### 2.5. Statistical Analysis

The sample size was calculated for the confidence level of 90% and margin of error of 10%. The sampling was applied in a model of so-called cluster-correlated data, as defined by Williams [33], that is described as a situation when samples within a cluster may be correlated, whereas there is no correlation between clusters [34]. In the applied model, a data defined as clustered are data from one respondent that is allowed in a medical studies for the data defined as not easily predictable (changeable during a course of disease) [35]. The ulcerative colitis and its symptoms is within this criteria due to well known changeable course of the disease [36]. Based on the indicated assumption, the calculated number of measurements was 43, so 44 measurements were planned for each sub-group, and time lapse between assessments of one patient was planned as 2 weeks, due to the fact that it is indicated as required time to observe diet-induced changes of haematological parameters [37]. The groups of 11 respondents in each sub-group were planned to be pair-matched, in order to obtain a higher reliability of the results.

The obtained data are presented as means ± standard deviations (SD) with minimum, maximum and median values. The distributions of the analyzed factors were verified by using the Shapiro-Wilk test. Differences between groups were identified by using the t-Student test (applied for parametric distribution) and the Mann-Whitney U test (applied for nonparametric distribution). Analysis of correlation was conducted using the Pearson correlation (applied for parametric distribution) and the Spearman rank correlation coefficient (applied for nonparametric distribution). The accepted level of significance was set at *p* ≤ 0.05. Statistical analysis was conducted using Statistica software version 8.0 (StatSoft Inc., Tulsa, OK, USA).

## 3. Results

The basic characteristics of the group of individuals with ulcerative colitis in remission phase recruited for the study in comparison with the pair-matched control group is presented in Table 1. It was stated that the assessed characteristics did not differ between groups.

The additional characteristics of the group of individuals with ulcerative colitis in remission phase recruited for the study is presented in Table 2.

The levels of chosen food products intake in ulcerative colitis group and control group are presented in Table 3. The group of ulcerative colitis individuals was characterized not only by higher intake of meat products (*p* < 0.001), but also by higher intake of meat products per 1000 kcal (*p* < 0.001), than a control group. In the case of intake of meat (*p* = 0.061) and intake of meat per 1000 kcal (*p* = 0.083), the differences were close to significance. The opposite relations were stated for the intake of rennet cheese (*p* = 0.001) and intake of rennet cheese per 1000 kcal of diet (*p* = 0.001), as the control group was characterized by higher intake than group of ulcerative colitis individuals. For other dairy products, no differences between groups were observed.

The levels of chosen nutrients intake in ulcerative colitis group and control group are presented in Table 4. The significant differences were stated only for intake of calcium (*p* = 0.003) and intake of calcium per 1000 kcal of diet (*p* = 0.001), as the control group was characterized by higher intake than group of ulcerative colitis individuals.

The levels of red blood cell count results in ulcerative colitis group and control group are presented in Table 5. In spite of the fact, that for RBC, HGB and HCT, no statistically significant differences between groups were observed, in the case of other parameters, a number of differences were stated between a group of ulcerative colitis individuals and a control group. The group of ulcerative colitis individuals was characterized by a lower values of MCV, MCH and MCHC, but simultaneously by a higher values of RDW, than a control group.

The analysis of association between chosen food products intake and red blood cell count results in ulcerative colitis group and control group is presented in Table 6. It was stated, that the associations in the ulcerative colitis group and control group were different. In spite of the fact, that a meat and meat products intake were correlated with a number of red blood cell count results in ulcerative colitis group, in a control group such associations were not stated. Moreover, in spite of a fact, that a negative correlation between dairy beverages intake and red blood cell count results was stated in a control group, in the case of ulcerative colitis group, the correlation was positive.

The analysis of association between chosen nutrients intake and red blood cell count results in ulcerative colitis group and control group is presented in Table 7. Similarly, as in the case of the analysis of food products intake, the correlations observed in analyzed groups differed. In the ulcerative colitis group it was stated, that the daily intake results of all the analyzed nutrients were positively correlated with red blood cell count results. In spite of the fact, that for animal protein, the association was similar, while recalculated per 1000 kcal, for iron and vitamin B_12_, while recalculated per 1000 kcal, the association was opposite in mentioned group. However, in the control group a number of associations were not observed-only the daily intake of vitamin B_12_ influenced red blood cell count results.

It was stated, that the sodium intake in a group of ulcerative colitis individuals correlated with the red blood cells (RBC) (*p* = 0.001; R = 0.5405; Pearson correlation), haemoglobin (HGB) (*p* = 0.001; R = 0.5214; Pearson correlation) and hematocrit (HCT) (*p* < 0.001; R = 0.5772; Spearman rank correlation coefficient).

**Figure 2:**
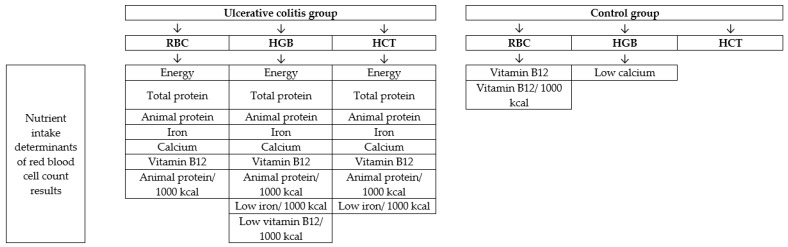
Nutrient intake determinants of red blood cell count results in ulcerative colitis group and control group. RBC-red blood cells; HGB-haemoglobin; HCT-hematocrit.

**Figure 3:**
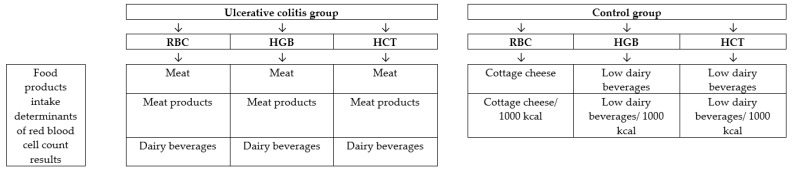
Food products intake determinants of red blood cell count results in ulcerative colitis group and control group.

The nutrient intake determinants of red blood cell count results in ulcerative colitis group and control group are presented in Figure 2 and the food products intake determinants of red blood cell count results in ulcerative colitis group and control group are presented in Figure 3.

## 4. Discussion

The main observations from the conducted study are the differences of noted associations between ulcerative colitis group and a control group. Not only the intake of food products and nutrients, as well as red blood cell count results differed between groups, but also the influence of intake on the red blood cell count results was stronger in the case of ulcerative colitis group, than in healthy ones.

The observed higher intake of meat and meat products, in the ulcerative colitis group, while dairy products intake was comparable or even lower, than in the control group, is confirmed by the studies of other authors. In the study of Rosman-Urbach et al. [38] it was observed, that in a group of ulcerative colitis individuals, the combined intake of meat and eggs was defined as 6 servings a day and it was significantly higher than in a control group, in which it was 4 servings a day. Such a high meat intake commonly contributes to a higher protein intake, than in the case of control individuals [39], however, it was not observed in own study.

On the other hand, not only high protein intake, but also a high meat and meat products intake is indicated as associated with increased risk of relapses [18] and, as a result, is currently indicated as a threat, that should be avoided [40]. However, the reasons of the negative influence of meat and meat products are unknown and a number of potential factors associated with meat products are indicated-the sulphur compounds [41], heterocyclic amines [42] and bacterial influence [43], so the impact of meat products may be multifactorial.

The potential advantage of high meat intake is associated with high content of highly available heme iron in meat products [44], however in the conducted own study, in spite of the higher meat products intake, ulcerative colitis individuals were characterized by similar iron intake as the control group. Such situation may result from the lower intake of non-heme iron from the plant products, than in the control group. It was also observed in the other studies, as in the study of Lomer et al. [45], the iron intake from cereals in the Crohn disease individuals was significantly lower than in the control group.

Such a higher proportion between heme and non-heme iron, than in healthy individuals, must be also considered as a factor that may increase the risk of relapses, as in animal-model study of Schepens et al. [46], it was stated, that the heme iron is the meat-related dietary factor, being responsible for the commonly stated negative influence of meat products. However, due to the lack of similar observations in the human studies, this hypothesis needs further verifications.

Simultaneously, in the conducted study, in the ulcerative colitis individuals, the lower rennet cheese intake was observed, and it contributed to the lower calcium intake, in comparison with the control group. Similar low dairy products intake is observed in a number of studies of inflammatory bowel disease patients, in comparison with a group of healthy individuals [47], that is associated inter alia with intentional limitation of dairy products intake, due to disease symptoms [48] and disease activity [49]. The consequence of the low calcium intake is the reduced bone mineral density [50], contributing to 40% higher fracture incidence in inflammatory bowel disease ones, than in the general population [51]. However, taking into account the anemia risk in ulcerative colitis individuals, the low calcium intake must be perceived as a factor that may increase iron absorption [31]. It may be stated, that if the meat intake in ulcerative colitis individuals is higher than in control ones, but the iron intake is comparable, as in the analyzed group, the relapses risk (due to high meat intake), accompanied by anemia risk (due to not higher iron intake), may be stated. However, the low calcium intake may reduce anemia risk and, at the same time, contribute to the increased osteoporosis risk.

The observed multiple positive correlations between meat and meat products, as well as dairy products intake and red blood cell count results were stated only in the case of ulcerative colitis group, and not for a control group. Such association between the food products intake and red blood cell count results were also observed in the other studies of inflammatory bowel diseases, as it was stated, that patients who restrict meat products have lower ferritin values [52].

Such a stronger association between food products intake and red blood cell count results, than in the control group may result from the higher susceptibility of red blood cell count results for the diet-influenced changes in the analyzed group than in healthy ones, that may result from higher iron needs, associated with both anemia of chronic disease (so called anemia of inflammation) and anemia of mixed origin [53]. Such a higher susceptibility of red blood cell count results for the diet-influenced changes is confirmed by the results of the study of Powell et al. [54], as it was concluded, that iron status is in inflammatory bowel disease individuals more closely related to the quality and quantity of dietary iron intake than in the general healthy population.

The management of the iron-deficiency anemia in inflammatory bowel diseases is indicated in the systematic review of Nielsen et al. [55] as an issue that causes many doubts for physicians, due to the need for the implementation of evidence-based recommendations taking into account the choice of the iron therapy in patients being intolerant to some therapy options, or having inadequate responses for some therapy options. Taking it into account, on the basis of the conducted study, the need for not only supplementation, but also the need for properly planned and conducted diet therapy, should be emphasized [56]. The diet therapy could be a good alternative option, especially as in a number of inflammatory bowel disease patients, the improperly planned iron supplementation is stated and it is indicated, that it may enhance free radicals generation and as a result, promote inflammation [57].

While compared the correlation with the red blood cell count results, observed for the daily nutritional value of diet and nutritional value of diet per 1000 kcal, in the inflammatory bowel disease group, the differences of results observed for iron seems to be the most important. The positive correlation for the daily iron intake accompanied by the negative correlation for the iron intake recalculated per 1000 kcal of diet, indicates, that the iron intake is an important factor, that must be taken into account, but only in the relation to the energy value of diet. It is confirmed by the general observations, that dietary iron intake is not an independent factor influencing anemia development, but a number of interfering factors must be taken into account [58], including energy intake [59]. It is also observed in the presented own study. The negative correlation for the iron intake per 1000 kcal of diet indicates, that the iron intake is beneficial, but only while accompanied by the high energy value of diet, and while it is accompanied by low energy value of diet (high iron intake per 1000 kcal), it is no longer beneficial.

In spite of the important observations indicated after comparison of a group of remission ulcerative colitis individuals and a group of pair-matched control individuals, after 6 weeks of constant observation, some limitations of the study must be mentioned. The study was conducted in a small group, as a number of 37 and 38 observations, for ulcerative colitis individuals and control individuals, respectively, were obtained. The future studies should be conducted in a larger groups in order to confirm the observations in more diverse patients. However, the following studies should be still conducted as a pair-matched model, in order to reliably compare observations for ulcerative colitis ones and healthy individuals.

Moreover, it would be beneficial to obtain in the future studies also the results of the other, than red blood cell count results, indicators of nutritional status. Such more complex analysis would enable assessment of the associations between nutrients intake, status and red blood cell count results and, in the future, practical formulation of the recommendations for ulcerative colitis patients.

## 5. Conclusions

In spite of the higher meat products intake in the group of ulcerative colitis individuals, in comparison with a pair-matched control group, the iron intake did not differ between groups, that may result from the higher intake of non-heme iron from plant products in the control group.

The positive correlations between meat and meat products intake, as well as total protein, animal protein, iron and vitamin B_12_ intakes and red blood cell count results were stated for group of ulcerative colitis individuals, while in the pair-matched control group were not observed, that may have resulted from higher susceptibility of red blood cell count results for the diet-influenced changes.

The positive correlation between red blood cell count results and energy value of diet, as well as daily iron intake observed in ulcerative colitis individuals, accompanied by negative correlation between red blood cell count results and iron intake recalculated per 1000 kcal of diet may indicate, that higher iron intake may be for mentioned patients beneficial, but only while accompanied by high energy value of diet.

It was stated, that diet of ulcerative colitis individuals must be monitored and modified, if needed, taking into account not only nutritional value of diet and products intake, but also the biochemical analysis results and declared symptoms, in order to reduce the risk of anemia and other extra-intestinal complications of the disease.

## Figures and Tables

**Table 1:** The basic characteristics of the group of individuals with ulcerative colitis in remission phase recruited for the study in comparison with the pair-matched control group.

		Ulcerative Colitis Group (*n* = 11)	Control Group (*n* = 11)	*p*-Value
Female Individuals		9 (81.8%)	9 (81.8%)	1.0000 ^a^
Male Individuals		2 (18.2%)	2 (18.2%)	
Age (years)		26.0 (20.0–56.0) *	25.0 (22.0–55.0) *	1.0000 ^b^
Body Mass (kg)		57.0 (41.0–124.0) *	59.0 (55.0–77.0) *	0.8438 ^b^
Height (cm)		171.0 (144.0–176.0) *	168.0 (156.0–183.0) *	0.7676 ^b^
Body Mass Index (BMI)	(kg/m^2^)	23.6 ± 6.2	22.2 ± 3.4	0.6936 ^c^
	Underweight	2 (18.2%)	1 (9.1%)	0.6551 ^a^
	Normal Body Mass	6 (54.5%)	8 (72.7%)	
	Overweight	2 (18.2%)	2 (18.2%)	
	Obesity	1 (9.1%)	0 (32.4%)	
Concurrent diseases **	D50-D64	1 (9.1%)	1 (9.1%)	1.0000 ^a^
	K00–K46; K65–K93	6 (54.5%)	6 (54.5%)	1.0000 ^a^
	L00–L99	3 (27.3%)	3 (27.3%)	1.0000 ^a^
	M00–M99	1 (9.1%)	1 (9.1%)	1.0000 ^a^

* nonparametric distribution (verified using the Shapiro-Wilk test; *p* < 0.05); ** based on International Statistical Classification of Diseases and Related Health Problems (ICD-10) [27], for: anaemias (D50–D89), diseases of the digestive system other than noninfective enteritis and colitis as well as other diseases of the intestines (K00–K46; K65–K93), diseases of the skin and subcutaneous tissue (L00–L99), diseases of the musculoskeletal system and connective tissue (M00–M99); ^a^ compared using chi^2^ test; ^b^ compared using Mann-Whitney U test based on median values (applied due to non-parametric distributions); ^c^ compared using t-Student test.

**Table 2:** The characteristics of the group of individuals with ulcerative colitis in remission phase recruited for the study.

		Ulcerative Colitis Group (*n* = 11)
Applied treatment	5-aminosalicylic acid medications alone	8 (72.7%)
	5-aminosalicylic acid medications combined with immunosuppressive ones	3 (27.3%)
Number of Bowel Movements per Day	1	4 (36.4%)
	2	6 (54.5%)
	≥3	1 (9.1%)
Erythrocyte Sedimentation Rate	<10	10 (90.9%)
	≥10	1 (9.1%)

**Table 3:** The levels of chosen food products intake in ulcerative colitis group and control group.

Intake	Food Products Group	Ulcerative Colitis Group (*n* = 37)	Control Group (*n* = 38)	*p*-Value **
		Median (Min.–Max.)	Median (Min.–Max.)	
Daily Intake	Meat	66.67 * (0–270.00)	33.33 * (0–220.00)	0.061
	Meat Products	40.00 (0–101.67)	0 * (0–130.00)	<0.001
	Dairy Beverages	80.00 * (0–225.00)	96.67 * (0–330.00)	0.274
	Cottage Cheese	0 * (0–116.67)	0 * (0–200.00)	0.629
	Rennet Cheese	10.00 * (0–66.70)	26.67 * (0–106.67)	0.001
Intake per 1000 kcal	Meat	34.37 (0–104.82)	23.16 * (0–108.51)	0.083
	Meat Products	21.67 (0–65.88)	0 * (0–76.11)	<0.001
	Dairy Beverages	41.92 * (0–134.56)	61.65 * (0–189.56)	0.157
	Cottage Cheese	0 * (0–49.01)	0 * (0–75.84)	0.617
	Rennet Cheese	5.78 * (0–47.74)	14.82 * (0–50.82)	0.001

* nonparametric distribution (verified using the Shapiro-Wilk test; *p* < 0.05). ** compared using Mann-Whitney U test (for nonparametric distribution).

**Table 4:** The levels of chosen nutrients intake in ulcerative colitis group and control group.

Intake	Nutrients	Ulcerative Colitis Group (*n* = 37)	Control Group (*n* = 38)	*p*-Value **
		Mean ± SD/Median (Min.–Max.)	Mean ± SD/Median (Min.–Max.)	
Daily Intake	Energy (kcal)	1814.9 ± 574.5	1763.5 ± 451.6	0.667
	Total Protein (g)	66.40 ± 20.69	64.99 ± 18.03	0.754
	Animal Protein (g)	40.72 ± 17.81	37.65 ± 14.05	0.410
	Iron (mg)	11.21 ± 3.19	11.54 ± 3.90	0.697
	Calcium (mg)	521.92 * (247.60–1154.90)	634.85 (324.90–1250.78)	0.003
	Vitamin B_12_ (µg)	3.03 * (0.50–16.90)	2.75 * (0.60–7.10)	0.803
Intake per 1000 kcal	Total Protein (g/1000 kcal)	37.15 ± 6.18	37.12 ± 6.26	0.982
	Animal Protein (g/1000 kcal)	22.14 ± 6.83	21.39 ± 6.22	0.620
	Iron (mg/1000 kcal)	5.95 *(4.22–10.95)	6.21 * (3.44–11.95)	0.699
	Calcium (mg/1000 kcal)	275.96 * (152.80–747.19)	377.69 (194.49–656.92)	0.001
	Vitamin B_12_ (µg/1000 kcal)	1.49 * (0.31–6.94)	1.56 * (0.56–3.94)	0.979

* nonparametric distribution (verified using the Shapiro-Wilk test; *p* < 0.05). ** compared using t-Student test (for parametric distribution) and Mann-Whitney U test (for nonparametric distribution).

**Table 5:** The levels of red blood cell count results in ulcerative colitis group and control group.

Blood Component	Ulcerative Colitis Group (*n* = 37)	Control Group (*n* = 38)	*p*-Value **
	Mean ± SD/Median (Min.–Max.)	Mean ± SD/Median (Min.–Max.)	
RBC (10^6^/dL)	4.52 ± 0.37	4.44 ± 0.26	0.290
HGB (g/dL)	13.40 (10.60–17.10)	13.80 * (12.40–15.20)	0.153
HCT (%)	40.70 * (34.50–48.70)	41.00 (35.30–46.40)	0.714
MCV (fL)	91.00 * (82.20–95.60)	92.50 * (85.00–97.60)	0.006
MCH (pg)	30.10 (25.70–34.70)	31.70 * (28.10–33.20)	<0.001
MCHC (g/dL)	33.30 * (30.70–36.60)	33.90 (32.70–35.10)	<0.001
RDW (%)	12.00 * (10.90–16.00)	11.50 (10.30–13.10)	0.001

* nonparametric distribution (verified using the Shapiro-Wilk test; *p* < 0.05). ** compared using t-Student test (for parametric distribution) and Mann-Whitney U test (for nonparametric distribution). RBC-red blood cells; HGB-haemoglobin; HCT-hematocrit; MCV-mean corpuscular volume; MCH-mean corpuscular hemoglobin; MCHC-mean corpuscular hemoglobin concentration; RDW-red blood cell distribution width.

**Table 6:** Analysis of association between chosen food products intake and red blood cell count results in ulcerative colitis group and control group.

Group	Intake	Blood Component	Meat		Meat Products		Dairy Beverages		Cottage Cheese		Rennet Cheese	
			*p*-Value	R	*p*-Value	R	*p*-Value	R	*p*-Value	R	*p*-Value	R
Ulcerative Colitis Group	Daily Intake	RBC	0.017 *	0.390	0.011	0.414	0.017 *	0.389	0.151 *	0.241	0.635 *	0.081
		HGB	0.006 *	0.445	0.012	0.409	0.032 *	0.353	0.143 *	0.246	0.396 *	0.144
		HCT	0.007 *	0.433	0.002 *	0.492	0.039 *	0.340	0.173 *	0.229	0.397 *	0.143
	Intake per 1000 kcal	RBC	0.298	0.176	0.252	0.193	0.118 *	0.262	0.273 *	0.185	0.906 *	−0.020
		HGB	0.200	0.216	0.492	0.117	0.230 *	0.202	0.259 *	0.190	0.864 *	0.029
		HCT	0.389 *	0.146	0.119 *	0.260	0.270 *	0.186	0.293 *	0.178	0.859 *	0.030
Control Group	Daily Intake	RBC	0.138 *	−0.245	0.926 *	0.015	0.355 *	−0.154	0.033 *	0.357	0.672 *	0.071
		HGB	0.599 *	−0.088	0.346 *	0.157	0.016 *	−0.390	0.062 *	0.306	0.492 *	−0.115
		HCT	0.688 *	−0.067	0.301 *	0.172	0.027 *	−0.359	0.110 *	0.264	0.826 *	−0.037
	Intake per 1000 kcal	RBC	0.056 *	−0.313	0.999 *	−0.000	0.258 *	−0.188	0.033 *	0.347	0.887 *	0.024
		HGB	0.453 *	−0.125	0.367 *	0.150	0.002 *	−0.479	0.068 *	0.299	0.426 *	−0.133
		HCT	0.548 *	−0.101	0.307 *	0.170	0.005 *	−0.443	0.121 *	0.256	0.779 *	−0.047

* analyzed using Spearman rank correlation coefficient for nonparametric distribution (parametricity verified using the Shapiro-Wilk test; *p* < 0.05); for parametric distribution analyzed using Pearson correlation; RBC-red blood cells; HGB-haemoglobin; HCT-hematocrit.

**Table 7:** Analysis of association between chosen nutrients intake and red blood cell count results in ulcerative colitis group and control group.

Group	Intake	Blood Component	Energy		Total Protein		Animal Protein		Iron		Calcium		Vitamin B_12_	
			*p*-Value	R	*p*-Value	R	*p*-Value	R	*p*-Value	R	*p*-Value	R	*p*-Value	R
Ulcerative Colitis Group	Daily Intake	RBC	<0.001	0.577	<0.001	0.687	<0.001	0.688	0.001	0.540	0.017 *	0.391	<0.001 *	0.706
		HGB	<0.001	0.701	<0.001	0.747	<0.001	0.748	0.001	0.526	0.011 *	0.413	<0.001 *	0.740
		HCT	<0.001 *	0.675	<0.001 *	0.741	<0.001 *	0.734	0.001*	0.514	0.012 *	0.409	<0.001 *	0.746
	Intake per 1000 kcal	RBC			0.370	0.152	0.008	0.429	0.158 *	−0.237	0.114 *	−0.264	0.338	−0.162
		HGB			0.806	0.042	0.008	0.427	0.043 *	−0.033	0.075 *	−0.296	0.020	−0.381
		HCT			0.626 *	0.083	0.003 *	0.477	0.038*	−0.342	0.060 *	−0.312	0.126 *	−0.256
Control Group	Daily Intake	RBC	0.399	0.141	0.177	0.223	0.159	0.233	0.233	0.198	0.898	0.021	0.005 *	0.443
		HGB	0.138 *	0.245	0.257 *	0.189	0.502 *	0.112	0.116 *	0.259	0.361 *	−0.152	0.481 *	0.118
		HCT	0.186	0.219	0.327	0.163	0.354	0.154	0.451	0.126	0.650	−0.076	0.410 *	0.138
	Intake per 1000 kcal	RBC			0.276	0.181	0.182	0.221	0.072 *	0.296	0.833	−0.035	0.019 *	0.380
		HGB			0.357 *	−0.154	0.360 *	−0.153	0.333 *	0.161	0.026 *	−0.360	0.955 *	−0.009
		HCT			0.576	−0.094	0.932	0.014	0.531 *	0.105	0.218	−0.204	0.736 *	0.056

* analyzed using Spearman rank correlation coefficient for nonparametric distribution (parametricity verified using the Shapiro-Wilk test; *p* < 0.05); for parametric distribution analyzed using Pearson correlation; RBC-red blood cells; HGB-haemoglobin; HCT-hematocrit.
